# Characteristics of patients with infective endocarditis undergoing surgery: a retrospective case series from Vietnam

**DOI:** 10.1515/med-2025-1328

**Published:** 2026-01-22

**Authors:** Nguyen Kim Anh, Vu Tri Thanh, Van Hung Dung, Nguyen Hoang Dinh

**Affiliations:** Department of Cardiovascular and Thoracic Surgery, School of Medicine, University of Medicine and Pharmacy at Ho Chi Minh City, Ho Chi Minh City, Vietnam; Thu Duc General Hospital, Ho Chi Minh City, Vietnam; Department of Cardiac Surgery, Heart Institute of Ho Chi Minh City, Ho Chi Minh City, Vietnam; University Medical Center Ho Chi Minh City, University of Medicine and Pharmacy at Ho Chi Minh City, Ho Chi Minh City, Vietnam; Department of Surgery, Pham Ngoc Thach University of Medicine, Ho Chi Minh City, Vietnam

**Keywords:** infective endocarditis, clinical characteristic, microbiology, case series

## Abstract

**Objectives:**

Infective endocarditis (IE) is a life-threatening infection of the inner lining of the heart resulting in severe complications such as valvular destruction and systemic embolism. Surgical intervention is often required to manage advanced cases. This study describes the clinical, paraclinical, and microbiological characteristics of patients with IE undergoing valve surgery at a tertiary cardiovascular centre in a developing country.

**Methods:**

This retrospective study analysed 176 patients with IE who underwent valve surgery at a single centre in Vietnam between January 2019 and December 2023.

**Results:**

The mean age of the cohort was 43.6 ± 15.9 years, with 62.5 % male patients and 13.5 % having a history of cardiac surgery. Mitral valve involvement was the most common lesion (94.9 %), predominantly presenting as regurgitation (96.4 %). The mean white blood cell count was 10.3 ± 8.6 K/μL, indicating an inflammatory response. Blood cultures were positive in 67.5 % of cases, with Viridans streptococci (34.6 %) and Streptococcus spp. (34.6 %) being the most frequently identified pathogens.

**Conclusions:**

This study provides comprehensive data on patients with IE undergoing surgery in Vietnam, highlighting the importance of early diagnosis, microbiological identification, and surgical intervention for improving patient outcomes in resource-limited settings.

## Introduction

Infective endocarditis (IE) is a severe and life-threatening endocardial infection primarily caused by bacteria or fungi. Despite advancements in diagnostic and therapeutic strategies, IE remains associated with significant morbidity and mortality, with in-hospital mortality rates ranging from 15 to 20 % and 1-year mortality approaching 40 % [1]. Severe complications, such as systemic embolism, heart failure, and intracardiac abscess, often necessitate timely surgical intervention to prevent irreversible structural damage.

IE can occur in patients with or without preexisting valvular disease [2]. Early surgical intervention during ongoing antibiotic treatment is considered in cases where it may help prevent progressive heart failure, irreversible structural damage from severe infection, and systemic embolism [3]. However, performing surgery during the active phase of the disease presents considerable risks. Advances in valve repair techniques, particularly for the mitral and tricuspid valves, have demonstrated the feasibility and efficacy of preserving native tissues that are more resistant to persistent or recurrent infections [4]. The feasibility of mitral valve repair for IE was first demonstrated by Dreyfus and Carpentier in 1990 [5]. Over the past decades, improvements in surgical techniques and accumulated clinical experience have contributed to favourable outcomes of mitral valve repair in patients with IE [6], 7].

Although IE has been widely studied, data regarding the clinical, paraclinical, and microbiological characteristics of patients undergoing surgical treatment remain limited, particularly in resource-limited settings. Understanding these characteristics is essential for improving disease management and optimising treatment strategies. This study aimed to provide a comprehensive description of the characteristics of patients with IE who underwent surgery at a single centre in Vietnam, contributing to the growing body of literature on IE and surgical outcomes in diverse healthcare settings.

## Methods

### Study setting and participants

This retrospective study was conducted at a specialised cardiovascular centre in Vietnam. All patients diagnosed with IE indicated for surgical intervention who underwent surgery at our institute between January 2019 and December 2023 were included in the study. This study was conducted in accordance with the Declaration of Helsinki and approved by the local ethics committee. Written informed consent was obtained from all patients for the publication of their data and accompanying images.

The timing of surgical intervention was based on the clinical severity and response to antibiotic therapy. In general, patients received a full course of intravenous antibiotics (typically 4–6 weeks) before surgery. However, in cases of worsening clinical conditions such as severe heart failure or uncontrolled infection, urgent or emergency surgery was performed before completion of the antibiotic regimen. All patients were monitored regularly through clinical examination and echocardiography to guide decision-making regarding surgical timing.

### Sample selection

Patients were selected based on a definite diagnosis of IE according to the modified Duke criteria and the presence of surgical indications as defined by the 2015 European Society of Cardiology (ESC) guidelines. All eligible patients who met these criteria and underwent valve surgery at our centre between January 2019 and December 2023 were included in the study. Patients were excluded if they met any of the following conditions: (1) IE involving non-valvular cardiac structures (e.g. isolated device-related IE); (2) incomplete medical records, including missing key diagnostic or operative data; (3) absence of follow-up data after discharge; and (4) non-bacterial causes of endocarditis (e.g. fungal or culture-negative cases without clinical confirmation).

### Data collection and statistical analysis

Data were extracted from the medical records of patients, including demographic information, clinical presentations, laboratory results, echocardiographic findings, microbiological data, and early postoperative outcomes. Postoperative outcomes included surgical timing, 30-day mortality, complications (pericardial effusion, pleural effusion, pneumonia, stroke), reoperations (for pericardial effusion, valve pathology, recurrent endocarditis, bleeding, or thrombus/vegetation), and ICU length of stay. Complications were classified according to the Clavien–Dindo grading system. Descriptive statistics were used to report outcome frequencies, percentages, and medians with interquartile ranges where appropriate.

The data were entered and cleaned using Excel and analysed using Stata version 13.0. Descriptive statistics were applied, and categorical variables were reported as frequencies and percentages. This work has been reported according to The PROCESS 2020 Checklist [8].

### Consent

Written informed consent was obtained from the patients for publication and any accompanying images. A copy of the written consent is available for review by the Editor-in-Chief of this journal on request.

## Results

### Participant characteristics

Table 1 presents the demographic and socioeconomic characteristics of the 176 patients, with a mean age of 43.6 ± 15.9 years. The majority of patients were male (62.5 %), whereas females accounted for 37.5 %. The most common age group was 40–59 years (43.8 %). Regarding nutritional status, 61.4 % of patients had a normal BMI (18.5–24.5), whereas 22.7 % were underweight (BMI<18.5) and 15.9 % were classified as overweight or obese (BMI>24.5).

**Table 1:** General characteristics of patients participating in the study (n=176).

Characteristic	Frequency	Ratio, %
**Age, years**	43.6 ± 15.9 (1–90)	
**Age group**		
<20	13	7.4
20–39	59	33.5
40–59	77	43.8
≥60	27	15.3
**Gender**		
Male	110	62.5
Female	66	37.5
**Nutritional status by BMI**		
Underweight (BMI<18.5)	40	22.7
Normal (BMI: 18.5–24.5)	108	61.4
Overweight (BMI>24.5)	28	15.9

### Clinical characteristics

Table 2 presents the clinical characteristics of patients, including underlying conditions and risk factors. Most patients were classified as NYHA grade II (46.0 %) or grade III (40.3 %). A total of 23 patients (13.5 %) had a history of cardiac intervention, with 94.9 % having no prior pneumonia. The most common comorbidities were hypertension (19.9 %) and diabetes (11.4 %). Preoperative atrial fibrillation and existing prosthetic valves were observed in 12.3 % of patients. Less common conditions included rheumatic heart disease, degenerative valve disease, mitral valve prolapse, and bicuspid aortic valves. Active infective endocarditis was documented in 83.0 % of cases, with 26.1 % classified as severe and 2.3 % presenting with CCS 4 chest pain. Respiratory failure was identified in 23.3 % of cases, primarily mild or moderate, whereas pulmonary hypertension was reported in 26.2 % of patients. In terms of surgical timing, 159 patients (90.3 %) underwent elective procedures, while 10 patients (5.7 %) required urgent intervention due to clinical deterioration, and 7 patients (4.0 %) underwent emergency surgery. The urgency of surgery was determined based on worsening heart failure or uncontrolled infection despite antibiotic therapy.

**Table 2:** Clinical characteristics of patients participating in the study (n=176).

Characteristic	Frequency	Ratio, %
NYHA classification		
I	12	6.8
II	81	46.0
III	71	40.3
IV	12	6.8
**History of cardiac intervention/Surgery**	23	13.5
**History of pneumonia**		
No	167	94.9
>30 d	7	4.0
<30 d	2	1.1
**Comorbidities**		
Hypertension	35	19.9
Diabetes	20	11.4
Dyslipidemia	15	8.5
Stroke	15	8.5
Peripheral artery disease	5	2.9
Chronic lung disease	1	0.6
**Cardiac conditions**		
Preoperative atrial fibrillation	21	12.3
Existing prosthetic valves	21	12.3
Ventricular septal defect	16	9.1
Degenerative valve disease	4	2.3
Bicuspid aortic valve	4	2.3
Atrial septal defect	2	1.2
Rheumatic heart disease	1	0.6
Mitral valve prolapse	1	0.6
Rupture of valsalva sinus	1	0.6
**Active infective endocarditis**	146	83.0
**Severe preoperative condition**	46	26.1
**CCS 4 chest pain**	**4**	**2.3**
**Pulmonary hypertension**		
No	130	73.9
Moderate	33	18.8
Severe	13	7.4
**Respiratory failure**		
No	135	76.7
Mild	23	13.1
Moderate	18	10.2
Severe	0	0.0
**Surgical urgency**		
Elective	159	90.3
Urgent	10	5.7
Emergency	7	4.0

### Paraclinical characteristics

Tables 3 and 4 summarise the clinical findings of the patients. Among the valvular lesions, mitral valve involvement was the most common (94.9 %), with regurgitation present in 96.4 % of cases and stenosis with regurgitation in 3.6 % of cases. Annular abscesses and ruptured chordae tendineae were observed in 3.6 and 20.4 % of cases, respectively. Aortic valve involvement was noted in 56.2 % of cases, with regurgitation being the predominant lesion (85.9 %). Tricuspid valve lesions were identified in 83.5 % of patients, whereas pulmonary valve involvement was rare (2.3 %), primarily presenting as severe stenosis with regurgitation. Vegetations were detected in 57.4 % of cases, with the majority measuring <10 mm (30.1 %).

**Table 3:** Echocardiographic and valvular characteristics of patients (n=176).

Characteristic		Frequency	Ratio, %
**Mitral valve lesions n=167 (94.9 %)**	**Type of lesion**		
	Regurgitation	161	96.4
	Stenosis + regurgitation	6	3.6
	**Annular abscess (yes)**	6	3.6
	**Ruptured chordae tendineae (yes)**	34	20.4
**Aortic valve lesions n=99 (56.2 %)**	**Type of lesion**		
	Regurgitation	85	85.9
	Stenosis + regurgitation	14	14.1
**Tricuspid valve lesions n=04 (2.3 %)**	**Type of lesion**		
	Severe stenosis + grade 3/4 regurgitation	2	50
	Grade 3/4 regurgitation + vegetation	1	25
	Vegetation	1	25

**Tricuspid valve lesions**		147	83.5

**Size of vegetations (n=176)**	No	75	42.6
	<10 mm	53	30.1
	10–15 mm	37	21.0
	15–30 mm	10	5.7
	>30 mm	1	0.6

**Table 4:** Laboratory and cardiac function parameters of patients (n=176).

Characteristic	n	Mean ± SD (min–max)
**Blood test**		
Hematocrit, %	176	33.2 ± 5.4 (18–55)
White blood cell count, k/μL	176	10.3 ± 8.6 (4–89)
Platelet count, k/μL	176	276 ± 115 (39–732)
Preoperative creatinine level, μmol/L	176	88.3 ± 34.5 (32–326)
Glomerular filtration rate, mL/min	174	88.2 ± 27.6 (22–178)
**Ejection fraction, %**	176	66.0 ± 8.9 (41–84)
**Left ventricular end-diastolic diameter, mm**	176	56.2 ± 8.9 (35–80)
**Left ventricular end-systolic diameter, mm**	176	35.4 ± 8.0 (18–63)
**Tapse, cm**	94	21.2 ± 7.3 (0–40)
**Left ventricular end-systolic diameter, mm**	167	46.3 ± 17.1 (22–110)

Regarding cardiac function, the mean ejection fraction was 66.0 ± 8.9 % (range: 41–84 %), remaining within normal limits. The mean left ventricular end-diastolic and end-systolic diameters were 56.2 ± 8.9 mm and 35.4 ± 8.0 mm, respectively. The mean TAPSE was 21.2 ± 7.3 mm, indicating severe right ventricular dysfunction in some patients (minimum value: 0 mm). The mean pulmonary artery systolic pressure was 46.3 ± 17.1 mmHg, reflecting a high prevalence of pulmonary hypertension.

Laboratory findings revealed that the mean haematocrit level was 33.2 ± 5.4 % (range: 18–55 %), whereas the mean white blood cell count was 10.3 ± 8.6 K/μL (range: 4–89 K/μL), consistent with an inflammatory or infectious process. The mean platelet count was 276 ± 115 K/μL (range: 39–732 K/μL). Preoperative renal function assessment showed a mean creatinine level of 88.3 ± 34.5 μmol/L (range: 32–326 μmol/L) and a mean glomerular filtration rate of 88.2 ± 27.6 mL/min (range: 22–178 mL/min).

### Microbiological characteristics of patients before surgery

Table 5 shows the microbiological findings of the patients. Blood cultures were performed in 87.5 % of cases, with 67.5 % yielding positive results. The most commonly identified pathogens were Viridans streptococci and Streptococcus spp., each accounting for 34.6 % of positive cases, followed by *Staphylococcus aureus* (15.4 %).

**Table 5:** Microbiological characteristics of patients before surgery (n=176).

Characteristic	Frequency	Ratio, %
**Blood culture (yes)**	154	87.5
**Positive culture results**		
Positive	104	67.5
Negative	50	32.5
**Microorganism identification results**		
*Viridans streptococci*	36	34.6
*Streptococcus spp*	36	34.6
*S. aureus without a primary source*	16	15.4
*Haemophilus spp*	3	2.9
*Actinobacillus actinomycetemcomitans*	2	1.9
*Eikenella spp*	2	1.9
*Kingella kingae*	2	1.9
*Cardiobacterium hominis*	1	1.0
*Coxiella burnetii*	1	1.0
Other microorganisms	24	23.1
**Description of other microorganisms**		
*Enterococcus spp*	6	24.0
*Abiotrophia defectiva*	4	16.0
*Staphylococcus coagulase* (−)	4	16.0
*Granulicatella adiacents*	3	12.0
*Stenotrophomonas maltophilia*	2	8.0
*Acinetobacter baumannii*	1	4.0
*Baccilus cenus*	1	4.0
*Rhodotorula spp*	1	4.0
*Candida*	1	4.0
*S. aureus from urine culture*	1	4.0

Rare pathogens from the HACEK group, including Haemophilus spp. and *Kingella kingae*, were identified in 1.0–2.9 % of cases. Enterococcus spp. were detected in 24 % of positive cultures. Additionally, uncommon microorganisms, such as *Abiotrophia defectiva* and Staphylococcus coagulase-negative bacteria, and fungal pathogens, such as Candida spp. and Rhodotorula spp., were observed.

Negative blood cultures were reported in 32.5 % of patients, highlighting the diagnostic challenges associated with early antibiotic administration or fastidious organisms.

### Early postoperative outcomes

Thirty-day mortality was 2.8 %. Postoperative complications occurred in 18.8 % of patients, including pericardial effusion (7.4 %), pleural effusion (5.7 %), pneumonia (1.1 %), and irreversible stroke (0.6 %). Reoperations were performed for pericardial effusion (2.3 %), valve pathology (2.3 %), recurrent endocarditis (2.3 %), bleeding (1.7 %), and thrombus or vegetation (1.1 %). Median ICU stay was 3 days (IQR 2–4).

## Discussion

This study provides valuable insights into the clinical, paraclinical, and microbiological characteristics of patients undergoing valve repair for IE in Vietnam. Conducted at the Heart Institute of Ho Chi Minh City, the study included a wide age range of patients (1–90 years), with the majority belonging to the working-age population, particularly 40–59 years (43.8 %) and 20–39 years (33.5 %). The mean age was 43.6 ± 15.9 years, notably lower than that in international studies. Awad et al. (2024) [9] reported mean ages of 55.2 ± 13.7 years and 57.8 ± 14.2 years for patients undergoing mitral valve repair and replacement, respectively, with age influencing recurrence and reoperation outcomes. Males accounted for 62.5 % of our cohort, a finding consistent with that of systematic reviews and long-term cohort studies. A systematic review by Shih et al. (2021) involving 271 patients with IE undergoing mitral valve surgery across the U.S., Germany, Belgium, Italy, and Taiwan also reported a male predominance of 60.1 % [10].

### Heart failure and comorbidities

The study revealed a predominance of heart failure classified as NYHA II (46.0 %) and NYHA III (40.3 %), indicating moderate-to-severe disease requiring clinical attention. Compared with developed nations such as the U.S., where NYHA III-IV rates were reported at 20.71 % by Slaughter et al. (2021) [11], and Germany, where El Gabry et al. (2019) [12] documented rates as high as 69 %, our findings highlight the exisiting variations in disease profiles and healthcare systems [12].

A history of prior cardiac intervention was recorded in 13.5 % of cases, which was lower than the 18 % reported in the systematic review by Shih et al. [10]. Differences in healthcare access, surgical techniques, and treatment approaches across regions may contribute to this discrepancy, emphasising the limited access to interventional treatments in Vietnam.

Hypertension (19.9 %) and diabetes mellitus (11.4 %) were the most common comorbidities. The prevalence of hypertension in our study was significantly lower than the 80.46 % reported by Nasso et al. (Italy) [13] but fell within the range of 18.04–26 % reported in other studies [10], 11], 14]. The prevalence of diabetes mellitus in our study aligned with those reported by Shih et al. [10] and Nasso et al. [13] but exceeded the 4.77 % observed by Slaughter et al. [11]. Because comorbidities influence treatment outcomes, these variations emphasise the need for tailored management strategies.

Preoperative atrial fibrillation was identified in 12.3 % of cases, which was lower than the 17.39–18.18 % reported by Xie et al. (2023). Given these findings, we propose individualised heart rate control strategies and anticoagulation protocols, carefully balancing thromboembolism risks with bleeding concerns during acute infections and urgent interventions [15].

### Valve lesions and surgical implications

Accurate data collection on valvular lesions is essential for optimising management strategies. Mitral valve involvement was the most prevalent (94.9 %), with regurgitation being the primary lesion (96.4 %). Complex pathologies included annular abscesses (3.6 %) and chordae tendineae rupture (20.4 %), further complicating disease severity and treatment decisions. Compared with the study by Tomšič et al. who reported 80 % severe mitral regurgitation in their repair cohort, our findings suggested differences in patient selection criteria and disease progression [7].

Aortic valve lesions were observed in 56.2 % of cases, predominantly presenting as regurgitation (85.9 %), although comparative data in similar populations remain limited. Tricuspid valve involvement was frequent (83.5 %), aligning with the 86 % prevalence reported by Di Mauro et al. (2023) [16]. Overall, mitral and tricuspid regurgitation emerged as the defining features of IE, reinforcing the need for timely surgical intervention and optimised perioperative management.

### Microbiological findings and diagnostic challenges

Active IE was diagnosed in 146 patients (83 %), with blood cultures performed in 87.5 % of cases, yielding a positivity rate of 67.5 %. The most commonly identified pathogens were V. streptococci and Streptococcus spp. (each 34.6 %), followed by *S. aureus* (15.4 %).

Rare HACEK group bacteria, including Haemophilus spp. and *K. kingae*, were identified in 1–2.9 % of cases, highlighting the diagnostic challenges associated with slow-growing organisms. Compared with other studies, Li et al. (2023) [17] reported a higher prevalence of *S. aureus* (27.8 %), with Slaughter et al. [11] reporting an even greater prevalence (77 %). Conversely, Folkmann et al. [18] and El Gabry et al. [12] reported Streptococcus spp. and *S. aureus* prevalence rates similar to or lower than those reported in the present study.

Notably, our study identified rare pathogens, including *A. defectiva*, coagulase-negative Staphylococcus, and fungal species, such as Candida spp. and Rhodotorula spp., highlighting the complex microbiology of IE. The 32.5 % rate of negative blood cultures further highlights the exisiting diagnostic challenges, which may be attributed to early antibiotic use or fastidious organisms. These findings reinforce the critical role of blood cultures and pathogen identification in optimising antibiotic regimens, particularly for complex or culture-negative infections.

Our surgical timing approach emphasised early infection control with antibiotic therapy. Among the 146 patients diagnosed with active IE, 138 (94.5 %) received a full course of antibiotics prior to surgery, in line with international recommendations. However, in a few cases, patients experienced clinical deterioration, such as decompensated heart failure or signs of uncontrolled infection despite ongoing antibiotic therapy. For these patients, urgent surgical intervention was required before completing the 4–6 week antibiotic course. These patients were closely monitored through clinical assessments and serial echocardiography to ensure the appropriate timing of surgery.

### Clinical implications and study limitations

Conducted at a specialised cardiovascular unit in southern Vietnam, this study provided a detailed analysis of the clinical, paraclinical, and microbiological characteristics of patients with IE undergoing valve surgery in the Vietnamese healthcare context. These insights support clinicians in making informed treatment decisions, optimising surgical timing, and reducing complications and mortality risks. These findings are highly applicable to clinical practice, particularly for managing IE in resource-limited settings.

In this study, elective surgery accounted for the majority of cases (90.3 %), with urgent and emergency procedures performed in 5.7 and 4.0 % of patients, respectively. This distribution reflects a predominantly stable patient population but also highlights the need for surgical readiness in managing decompensated cases. Timely intervention remains critical, particularly in resource-limited settings where delays may increase mortality and complication risks [19].

Early postoperative outcomes showed a 30-day mortality rate of 2.8 %, which is comparable to or lower than rates reported in similar surgical series. The overall complication rate was 18.8 %, primarily involving pericardial and pleural effusion. The need for reoperation, most commonly due to recurrent endocarditis or pericardial effusion, remained low. The median ICU stay was 3 days, consistent with expected postoperative recovery periods for valvular surgery. These findings support the feasibility and safety of surgical treatment for infective endocarditis in appropriately selected patients, even in a developing country setting [20].

However, this study had several limitations. First, it focused solely on surgical intervention without comparing outcomes with non-surgical (medical) management. Second, as this was a single-centre study, its findings may not be generalisable to a broader population or other healthcare settings. Future research should incorporate multi-centre studies and long-term follow-up data to enhance the applicability of these findings.

## Conclusions

This study provided a comprehensive overview of the clinical and microbiological characteristics of patients undergoing surgical treatment for IE in Vietnam. Mitral and tricuspid regurgitations were the most prevalent valve lesions and served as key surgical indicators.

Microbiological analysis identified V. streptococci, Streptococcus spp., and *S. aureus* as the primary pathogens; rare bacteria and fungi were also detected. The high rate of negative blood cultures highlights the diagnostic challenges.

These findings highlight the importance of surgical intervention in IE management and provide valuable insights for optimising treatment strategies and improving patient outcomes.
